# Factors That Predict Endoscopic Evaluation and Gastrostomy Placement in Patients With Neurologic Disorders and Dysphagia

**DOI:** 10.7759/cureus.88853

**Published:** 2025-07-27

**Authors:** R. Christopher Chase, Pedro Cortes, Christopher J Lamb, Dawn Francis, Kenneth R DeVault, Maoyin Pang

**Affiliations:** 1 Department of Internal Medicine, Mayo Clinic, Jacksonville, USA; 2 Department of Neurology, Mayo Clinic, Jacksonville, USA; 3 Department of Gastroenterology and Hepatology, Mayo Clinic, Jacksonville, USA

**Keywords:** amyotrophic lateral sclerosis, dysphagia, endoscopy, gastrostomy, neurologic disorders

## Abstract

Background: Although patients with neurologic disorders commonly develop dysphagia, there remains little consensus on the role of initial esophagogastroduodenoscopy (EGD) or temporal guidance on gastrostomy placement. We aimed to characterize the predictors associated with EGD and gastrostomy recommendation at the initial gastroenterology consultation in patients with progressive neurologic disorders.

Methods: This retrospective study spanned from December 31, 2010, to December 31, 2021, and included patients with both dysphagia and neurologic disorders. Multivariate logistic regression determined the predictors for EGD and gastrostomy recommendation after the initial visit.

Results: Out of 273 patients, EGD was recommended for 163 (59.7%) at the initial evaluation. A diagnosis of amyotrophic lateral sclerosis (ALS) (odds ratio: 0.20; 95% confidence interval (CI): 0.07-0.52; P=0.001) and being referred by a neurologist (odds ratio: 0.37; 95% CI: 0.17-0.84; P<0.02) were the negative predictors of an initial EGD recommendation. Gastrostomy was recommended for 38 patients (13.9%) at the initial consultation. Dysphagia to both liquids and solids, body mass index, diagnosis of ALS, and clinical frailty scale scores were associated with gastrostomy (P≤0.01). A model of six variables had high predictive accuracy for EGD recommendation (area under the receiver operating characteristic curve: 0.7741).

Conclusions: This study proposes a predictive model for initial EGD recommendation. We suggest that when considered in conjunction with the predictive clinical features of ALS diagnosis and dysphagia to both solids and liquids, the clinical frailty scale and American Society of Anesthesiologists physical status classification system scores may help clinicians anticipate gastrostomy when applied to patients with any neurologic disorder.

## Introduction

Neurologic disorders (NDs) cause dysphagia in 400,000-800,000 people worldwide each year [1]. This results in a high level of morbidity and a heavy cost on the US healthcare system. Dysphagia, defined as difficulty in swallowing liquid or food boluses, has been estimated to cost $4.3-7.1 billion each year in the United States [2]. The burden on the healthcare system is associated with the complexity of dysphagia management and investigation of the root cause. Dysphagia results in a significantly increased hospital length of stay, an increase in hospital stay costs by $6,243 on average, and a significantly higher frequency of transfers to post-acute care facilities [2].

History as well as an understanding of the underlying neurologic pathophysiology is key in the initial workup when determining whether the dysphagia is oropharyngeal or esophageal in nature [3]. Oropharyngeal dysphagia is typically identified by a history of food sticking or getting stuck in the throat. Aside from the well-known effects of hemorrhagic and ischemic stroke, dysphagia has been shown to be highly associated with NDs such as amyotrophic lateral sclerosis (ALS), multiple sclerosis (MS), and myasthenia gravis (MG) [3-6]. Different NDs have been shown to have their own dysphagia phenotype, ranging from oropharyngeal to esophageal [7-9]. Esophageal disorders may be represented by a physical abnormality or change in motility and frequently require further investigation with imaging and direct visualization. Imaging modalities include modified barium swallow (videofluoroscopic swallow study), high-resolution pharyngeal manometry, esophageal manometry, and esophagogastroduodenoscopy (EGD). Direct visualization with EGD is an essential diagnostic step in the workup of dysphagia of suspected esophageal origin given its diagnostic and therapeutic modality [4,10]. Despite this, there remains little consensus on the role of EGD in dysphagia secondary to ND.

The diagnostic dilemma of dysphagia reaches a higher degree of complexity when secondary to a neurologic etiology. NDs often occur with insidious onset, affecting multiple organ systems at a time. With the recent conceptualization of the brain-gut axis within the medical literature, there is a growing imperative to comprehend and predict the impact of NDs on the function of the gastrointestinal tract, encompassing both the nervous system and cognitive perspectives [11]. Accordingly, most patients with NDs otherwise do not complain of symptoms suggesting a digestive or absorptive abnormality. In such patients, a nasogastric tube (NGT) may be placed for enteral nutrition and oral medication administration. However, patients with continued dysphagia over a period of 30 days benefit from percutaneous endoscopic gastrostomy (PEG) tube placement. PEG is considered a safe method of enteric nutrition in this population [12-14]. Although there is no universally recognized time duration triggering a recommendation to switch from NGT to PEG, NGT is not recommended as a long-term solution due to a higher risk of complications, potential discomfort, inadequate stability, and negative impact on quality of life when compared to more suitable alternatives like gastrostomy or jejunostomy tubes. The long-term superiority of PEG over NGT was demonstrated in a Cochrane review of 11 randomized clinical trials that showed PEGs fail less than NGTs in delivering enteral nutrition [15]. Despite the common need for PEG in patients with NDs, there remains a gap in the medical literature exploring the predictors for the indication and appropriate timing of PEG tube placement [16].

We aimed to better characterize the predictors associated with the recommendation for EGD at the initial gastroenterology consultation of patients who were referred for dysphagia secondary to progressive ND, as well as the predictors for gastrostomy tube placement in this population.

## Materials and methods

Patient selection

This retrospective study was approved and deemed exempt by the Institutional Review Board at the Mayo Clinic in Florida. We searched the medical records of four academic tertiary care centers, namely, Mayo Clinic in Florida, Mayo Clinic in Rochester, Mayo Clinic in Arizona, and Mayo Clinic Health System, to identify patients with dysphagia and underlying neurologic or immune-mediated myopathy (i.e., ND) by using the International Classification of Diseases, Tenth Revision, Clinical Modification codes. Patients with ALS, lateral sclerosis, MG, MS, hereditary neuropathy, hereditary myopathy, critical illness myopathy, inflammatory myositis (e.g., dermatomyositis or polymyositis), and inclusion body myositis were included in the study if they also carried an inpatient or outpatient diagnosis of dysphagia. Inclusion criteria also required age over 18 and consultation with a gastroenterologist whether inpatient or outpatient for dysphagia. We retrospectively reviewed the medical records to identify whether the patient had a consultation with a gastroenterologist for dysphagia, termed the initial evaluation of dysphagia, between December 31, 2010, and December 31, 2021. Patients were excluded from the study for the following reasons: (1) age less than 18 years; (2) no diagnosis of an ND; (3) absence of dysphagia; and (4) lack of a consultation with a gastroenterologist for dysphagia.

Variable selection and primary outcome measures

We collected data retrospectively. Demographics included age at the initial evaluation, sex (male or female), race, body mass index (BMI), smoking history, and presence of moderate alcohol use (defined by National Institutes of Health criteria as >14 drinks/week for men and >7/week for women). Clinical data included the following: type of ND; time with disorder as measured from the date of the initial evaluation of dysphagia; American Society of Anesthesiologists (ASA) physical status classification system [17], clinical frailty scale (CFS) [18], and Charlson comorbidity index scores [19]; presence of arm or leg weakness; performance of an electromyogram; history of aspiration pneumonia, diabetes, or obstructive sleep apnea; use of positive airway pressure therapy or a portable ventilator at home; and performance of pulmonary function tests. Given the low number of patients with lateral sclerosis, hereditary myopathy, and critical illness myopathy, these disorders were grouped as other conditions. Data on the initial encounter included the type of dysphagia reported by the patient and the specialty of the referring clinician.

The first primary outcome was defined as whether an EGD was recommended at the initial encounter of dysphagia. Secondary outcomes included findings on EGD, endoscopic interventions performed at the time of EGD, performance of esophageal biopsies, whether an esophagram or manometry was recommended, and their respective findings.

The second primary outcome was defined as the placement of a gastrostomy (or jejunostomy) tube for feeding or medication administration. Gastrostomy placement methods included endoscopic, percutaneous, and surgical approaches. Time zero was defined as the date of the initial evaluation of dysphagia. Patients were censored at the time of the primary outcome, at the time of death, or at the time of last encounter within our hospital system.

Statistical analysis

Baseline characteristics were summarized with descriptive statistics. Continuous and categorical variables were reported as median with interquartile range (IQR) and number and percentage of patients, respectively. Comparison of baseline characteristics was performed using nonparametric tests, including the Wilcoxon rank sum and Fisher's exact tests. Logistic regression analysis was performed for the primary outcome to identify predictors, and odds ratios (ORs) and 95% confidence intervals (CIs) were estimated. Multivariable logistic regression was performed by adjusting for significant covariates and variables with biologic plausibility to affect the primary outcome, including age and sex classification. Categorical variables that occurred in less than 10 patients in the entire cohort were not examined in association analysis. The area under the receiver operating characteristic curve was determined for the multivariable model with the best discrimination for the primary outcome. Survival analyses, including Cox proportional hazards regression and Kaplan-Meier estimates, were used for the primary endpoint. Multivariable Cox regression analysis was performed to adjust for significant predictors, and hazard ratios (HRs) and 95% CIs were estimated. All statistical tests were two-sided. A P-value of less than 0.05 was considered statistically significant. The Bonferroni correction was not used given the exploratory nature of this study; therefore, a 5% chance of type 1 error was accepted. Statistical analysis was performed using IBM SPSS Statistics for Windows, V. 28.0 (IBM Corp., Armonk, NY, USA).

## Results

Baseline characteristics and outcomes

Table 1 summarizes the baseline characteristics of the 273 patients included in the study. The most common NDs were MS, MG, and ALS. Median IQR time with the disorder was 732 (−8.5-3,960) days by the initial evaluation of dysphagia. Most patients had solid dysphagia, followed by dysphagia to both solids and liquids. Median (IQR) CFS, ASA classification, and Charlson comorbidity index scores were 4 (3-5), 3 (2-3), and 3 (1-4), respectively.

**Table 1:** Patient baseline characteristics ^a^Other conditions included lateral sclerosis (n=1), hereditary myopathy (n=8), and critical illness myopathy (n=6). Data reported as No. (%) unless otherwise indicated. IQR: interquartile range; ASA: American Society of Anesthesiologists; BMI: body mass index; CCI: Charlson comorbidity index; CFS: clinical frailty scale; EGD: esophagogastroduodenoscopy; EMG: electromyography; ND: neurologic disorder; PAP: positive airway pressure; PFT: pulmonary function test

Demographics	All patients (n=273)	EGD not recommended (n=110)	EGD recommended (n=163)	P-value
Age at evaluation, median (IQR), year	63.9 (56.3-72.8)	68.0 (56.4-75.5)	63.2 (56.3-71.0)	0.06
70-79 years	67 (24.5)	35 (31.8)	32 (19.6)	0.03
Sex, male	116 (42.5)	54 (49.1)	62 (38.0)	0.08
Race, White	250 (91.6)	103 (93.6)	147 (90.2)	0.38
Smoking, active	12 (4.4)	5 (4.5)	7 (4.3)	>0.99
Alcohol use, moderate	23 (8.4)	10 (9.1)	13 (8.0)	0.83
BMI, median (IQR), kg/m^2^	27.8 (23.6-31.6)	26.5 (23.2-30.8)	28.4 (24.2-32.8)	0.16
Site of evaluation, Florida	43 (15.8)	12 (10.9)	31 (19.0)	0.09
Amyotrophic lateral sclerosis	42 (15.4)	32 (29.1)	10 (6.1)	0.01
Myasthenia gravis	61 (22.3)	17 (15.5)	44 (27.0)	0.03
Other conditions^a^	15 (5.5)	7 (6.4)	8 (4.9)	0.60
Hereditary neuropathy	39 (14.3)	15 (13.6)	24 (14.7)	0.86
Inflammatory myositis	22 (8.1)	10 (9.1)	12 (7.4)	0.65
Inclusion body myositis	20 (7.3)	7 (6.4)	13 (8.0)	0.81
Multiple sclerosis	74 (27.1)	22 (20.0)	52 (31.9)	0.04
CFS score, median (IQR)	4 (3-5)	4 (3-6)	3 (3-5)	0.01
ASA physical status classification system score, median (IQR)	3 (2-3)	3 (2-3)	3 (2-3)	0.05
CCI score, median (IQR)	3 (1-4)	3 (2-4)	3 (1-4)	0.29
EMG performed	120 (44.0)	55 (50.0)	65 (39.9)	0.11
Arm weakness	107 (39.2)	56 (50.9)	51 (31.3)	0.01
Leg weakness	105 (38.5)	52 (47.3)	53 (32.5)	0.02
Aspiration pneumonia	49 (17.9)	22 (20.0)	27 (16.6)	0.51
Speech swallow evaluation	99 (36.3)	48 (43.6)	51 (31.3)	0.041
Obstructive sleep apnea	105 (38.5)	41 (37.3)	64 (39.3)	0.80
PAP therapy	78 (28.6)	31 (28.2)	47 (28.8)	>0.99
Portable ventilator	15 (5.5)	13 (11.8)	2 (1.2)	0.01
PFTs performed	78 (28.6)	40 (36.4)	38 (23.3)	0.02
Normal PFTs	35 (44.9)	20 (50.0)	15 (39.5)	0.49
Diabetes	51 (18.7)	18 (16.4)	33 (20.2)	0.44
Time with ND, median (IQR), days	732.0 (−8.5-3,960.0)	290.0 (−7.5-2,225.5)	1,136.0 (−6.5-5,458.0)	0.03
Type of dysphagia				<0.05
Solids only	179 (65.6)	63 (57.3)	116 (71.2)	
Liquids only	8 (2.9)	5 (4.5)	3 (1.8)	
Both	86 (31.5)	42 (38.2)	44 (27.0)	
Referring clinician				
Neurologist	43 (15.8)	30 (27.3)	13 (8.0)	<0.01
Primary care physician	69 (25.3)	28 (25.5)	41 (25.2)	>0.99

Of the 163 patients recommended to undergo EGD, only 134 underwent the procedure. Table 2 summarizes the primary and secondary outcomes measured. Among patients who underwent an EGD, 68 (50.7%) had abnormal findings. The most common findings were strictures and reflux esophagitis. Endoscopic intervention was performed in 36 patients (26.9%), 28 of whom underwent esophageal dilatation.

**Table 2:** Outcomes of patients at the initial evaluation of dysphagia Data reported as No. (%). EGD: esophagogastroduodenoscopy; EGJ: esophagogastric junction

Outcomes	N=273
EGD recommended	163 (59.7)
EGD performed	134 (82.2)
Abnormal	68 (50.7)
Esophageal candidiasis	2 (2.9)
Reflux esophagitis	21 (30.9)
Eosinophilic esophagitis	6 (8.8)
Stricture	23 (33.8)
Other	40 (58.8)
Intervention performed	36 (26.9)
Esophageal dilatation	28 (77.8)
Food bolus removal	6 (16.7)
Biopsy performed	62 (46.3)
Barium swallow test recommended	89 (32.6)
Performed	75 (84.3)
Abnormal	42 (56.0)
Aspiration	9 (21.4)
Esophageal manometry recommended	32 (11.7)
Performed	24 (75.0)
Abnormal	18 (75.0)
EGJ outflow obstruction	2 (11.1)
Ineffective motility	16 (88.9)

**Figure 1:**
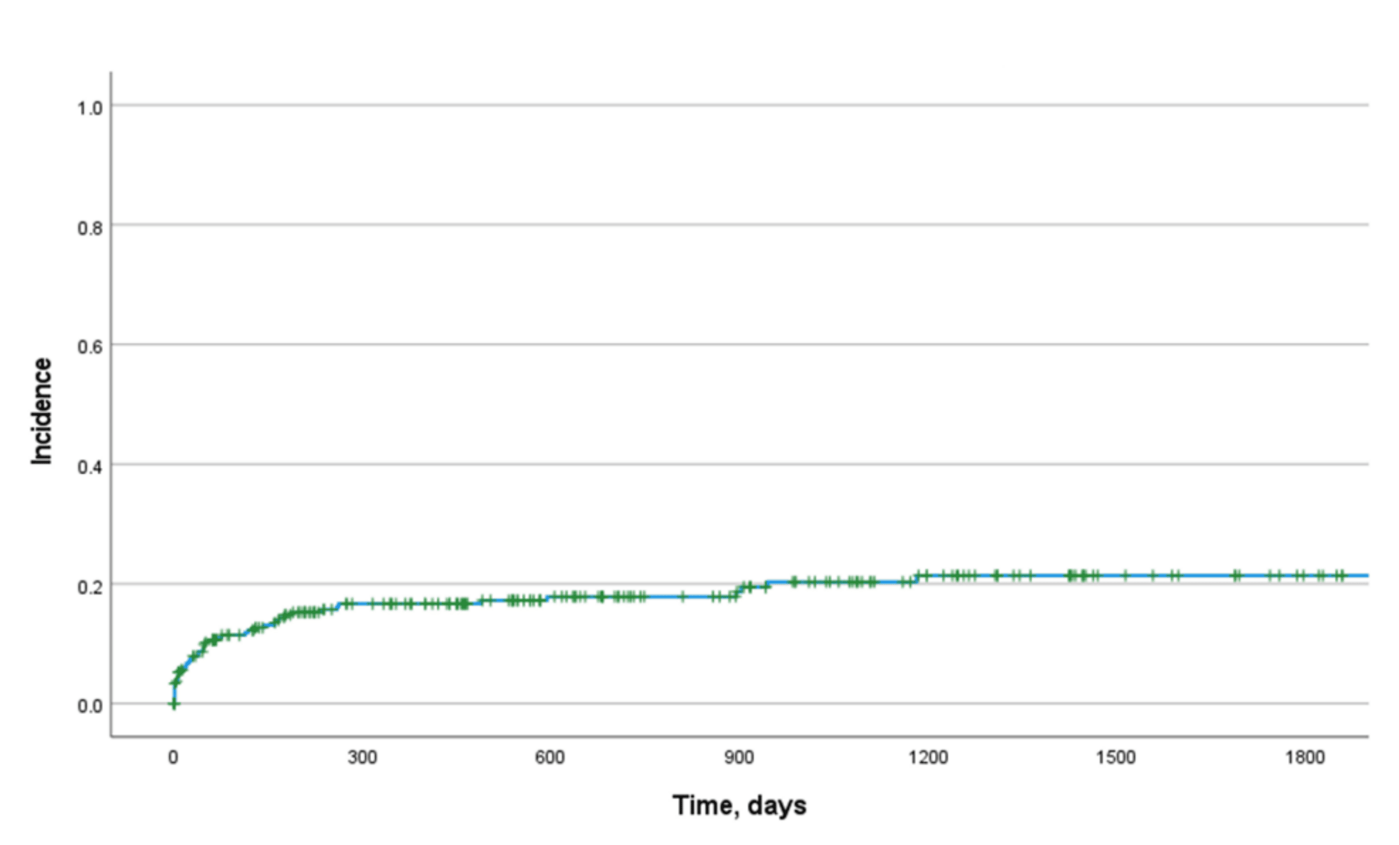
Cumulative incidence of gastrostomy placement The Kaplan-Meier curve of the incidence of gastrostomy placement for all patients.

A total of 51 patients had a gastrostomy tube placed at follow-up (Table 3). At the initial evaluation of dysphagia, 38 patients (13.9%) were recommended to have a gastrostomy, 32 of whom underwent placement. The median (IQR) time to gastrostomy placement for all patients was 48 (7-181) days (Figure 1).

**Table 3:** Primary endpoint of gastrostomy placement Data reported as No. (%) unless otherwise indicated.

Endpoints	N=273
Gastrostomy recommended after initial consultation	38 (13.9)
Performed	32 (84.2)
Not performed	6 (15.8)
Gastrostomy not recommended after initial consultation	235 (86.1)
Performed at follow-up	19 (8.1)
Patients with gastrostomy performed	51 (18.7)
Overall time to gastrostomy placement, median (IQR), days	48 (7-181)

Associations with EGD recommendation

In univariable analysis, having an EGD recommended was associated with multiple variables (Table 1). The strongest associations for an EGD not being recommended were a diagnosis of ALS (29.1% vs 6.1%; P<0.001), CFS score (4 vs 3; P<0.001), use of a portable ventilator at home (11.8% vs 1.2%; P<0.001), and being referred by a neurologist (27.3% vs 8%; P<0.001).

In unadjusted logistic regression, the strongest negative predictors for an EGD being recommended included a diagnosis of ALS (OR: 0.16; P<0.0001), CFS score (OR: 0.73 per 1 point; P<0.0001), and being referred by a neurologist (OR: 0.20; P<0.0001). Age, male sex, BMI, and history of aspiration pneumonia were not significant predictors (Table 4).

**Table 4:** Logistic regression analysis for EGD being recommended at the initial evaluation for dysphagia ^a^A total of 163/273 patients were recommended to undergo an EGD at evaluation by the consulting gastroenterologist. Given the outcome with the lowest event rate (n=110), the maximum number of variables allowed in the multivariable models was 11. Multivariable logistic regression models were adjusted for the strongest variables associated with an EGD being recommended at P<0.05 on univariable logistic regression. To avoid overfitting, models of six variables were constructed and estimated ORs are listed above. ^b^Six-variable multivariable models were constructed. The baseline model was adjusted for five variables, including male sex, age, and the three strongest variables: amyotrophic lateral sclerosis, being referred by a neurologist, and CFS score. ASA: American Society of Anesthesiologists; BMI: body mass index; CCI: Charlson comorbidity index; CFS: clinical frailty scale; EGD: esophagogastroduodenoscopy; EMG: electromyography; NA: not applicable; ND: neurologic disorder; OR: odds ratio; PFT: pulmonary function test

Demographics	Univariable		Multivariable^a^	
	OR (95% CI)	P-value	OR (95% CI)	P-value
Age at evaluation, per 1 year	0.98 (0.96-1.00)	0.13	1.00 (0.98-1.03)	0.91^b^
Sex, male	0.64 (0.39-1.05)	0.08	0.99 (0.52-1.89)	0.97^b^
Race, White	0.62 (0.25-1.57)	0.32	0.43 (0.12-1.50)	0.19
BMI, per 1 kg/m^2^	1.03 (0.99-1.07)	0.13	1.00 (0.95-1.04)	0.85
Site of evaluation, Florida	1.92 (0.94-3.92)	0.07	1.61 (0.66-3.93)	0.30
Amyotrophic lateral sclerosis	0.16 (0.08-0.34)	<0.01	0.20 (0.07-0.52)	<0.01^b^
Myasthenia gravis	2.02 (1.09-3.77)	<0.03	1.73 (0.75-3.99)	0.20
Hereditary neuropathy	1.09 (0.55-2.19)	0.80	0.60 (0.26-1.38)	0.23
Multiple sclerosis	1.87 (1.06-3.32)	0.03	0.90 (0.43-1.92)	0.79
CFS score, per 1 point	0.73 (0.63-0.86)	<0.01	0.85 (0.70-1.03)	0.09^b^
ASA physical status classification system score, per 1 point	0.64 (0.43-0.96)	<0.03	1.30 (0.72-2.35)	0.38
CCI score, per 1 point	0.96 (0.86-1.07)	0.47	1.00 (0.92-1.22)	0.99
Arm weakness	0.44 (0.27-0.72)	0.01	1.15 (0.55-2.42)	0.71
Leg weakness	0.54 (0.33-0.88)	0.01	0.90 (0.45-1.81)	0.76
EMG performed	0.66 (0.41-1.08)	0.01	1.11 (0.58-2.11)	0.76
Aspiration pneumonia	0.79 (0.43-1.48)	0.47	1.69 (0.70-4.11)	0.25
Speech swallow evaluation	0.59 (0.36-0.97)	<0.04	1.09 (0.55-2.16)	0.81
Portable ventilator	0.77 (0.02-0.42)	<0.01	0.46 (0.08-2.60)	0.38
PFTs performed	0.53 (0.31-0.91)	0.02	0.90 (0.44-1.83)	0.77
Diabetes	1.30 (0.69-2.44)	0.42	0.69 (0.31-1.52)	0.36
Time with ND, per 365 days	1.05 (1.02-1.08)	<0.01	1.04 (0.99-1.09)	0.08
Type of dysphagia				
Solids only	1.00 (reference)	NA	1.00 (reference)	NA
Liquids only	0.33 (0.08-1.41)	0.13	0.27 (0.06-1.29)	0.10
Both	0.57 (0.34-0.96)	0.03	0.58 (0.29-1.14)	0.29
Referred by a neurologist	0.20 (0.10-0.43)	<0.01	0.37 (0.17-0.84)	<0.02^b^

After adjusting for age, male sex, diagnosis of ALS, being referred by a neurologist, and CFS score in multivariable analysis, only two predictors remained significant (Table 4). A diagnosis of ALS was associated with 80% reduced odds of an EGD recommendation. Similarly, referral by a neurologist was associated with 63% reduced odds. Notably, the presence of dysphagia to liquids or to both solids and liquids was not associated with an EGD being recommended relative to having dysphagia to solids only.

**Figure 2:**
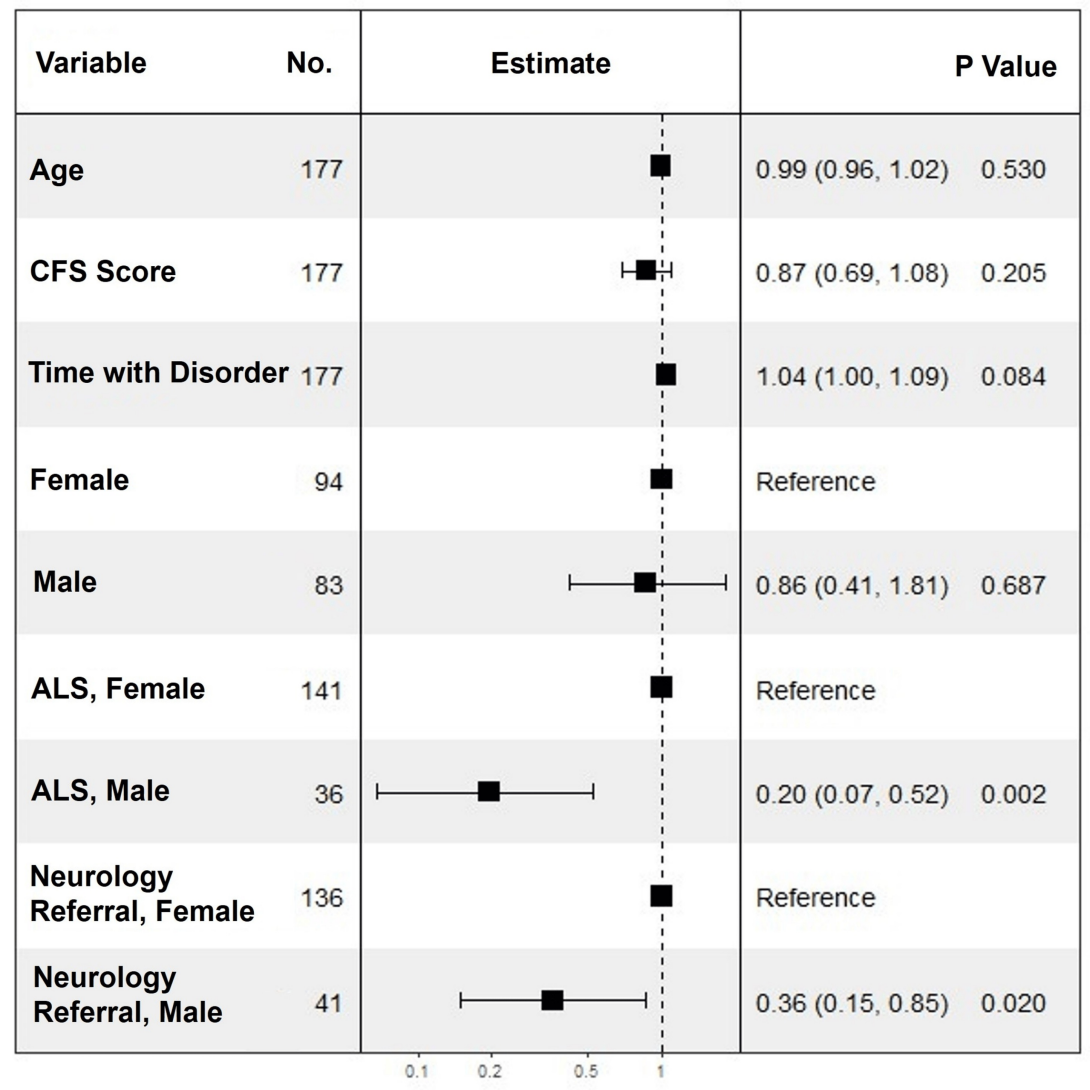
Forest plot of multivariable logistic regression model to predict esophagogastroduodenoscopy recommendation Age per one year, clinical frailty scale score per 1 point, and time with disorder per one year. CFS: clinical frailty scale; ALS: amyotrophic lateral sclerosis

**Figure 3:**
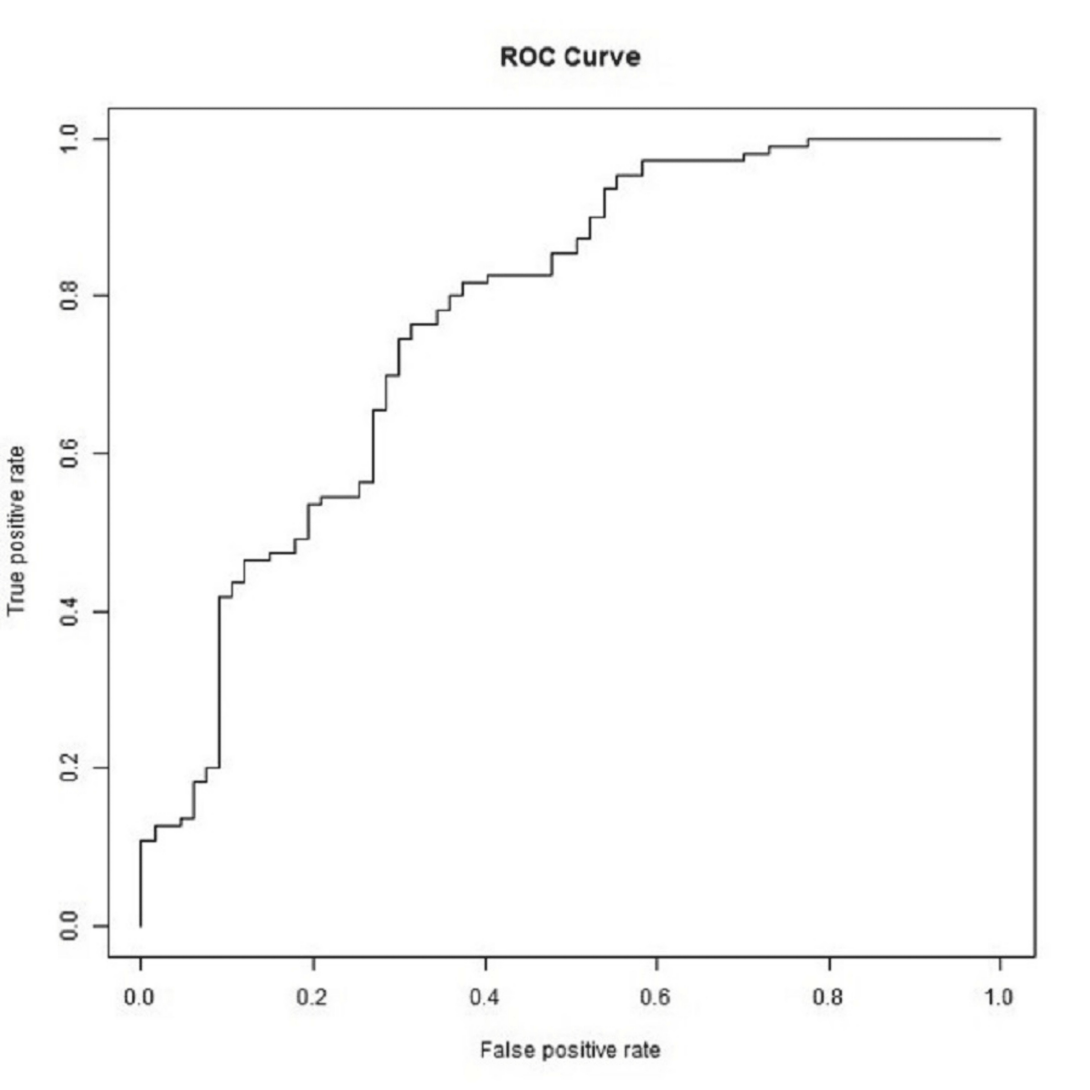
ROC curve of multivariable model for esophagogastroduodenoscopy recommendation Area under the ROC curve: 0.7741. ROC: receiver operating characteristic

A six-variable model, including age, CFS score, time with disorder, male sex, diagnosis of ALS, and being referred by a neurologist, had a high predictive accuracy (area under the receiver operating characteristic curve: 0.7741) for having an EGD recommended at the initial evaluation of dysphagia (Figure 2 and Figure 3).

Associations with gastrostomy placement

**Figure 4:**
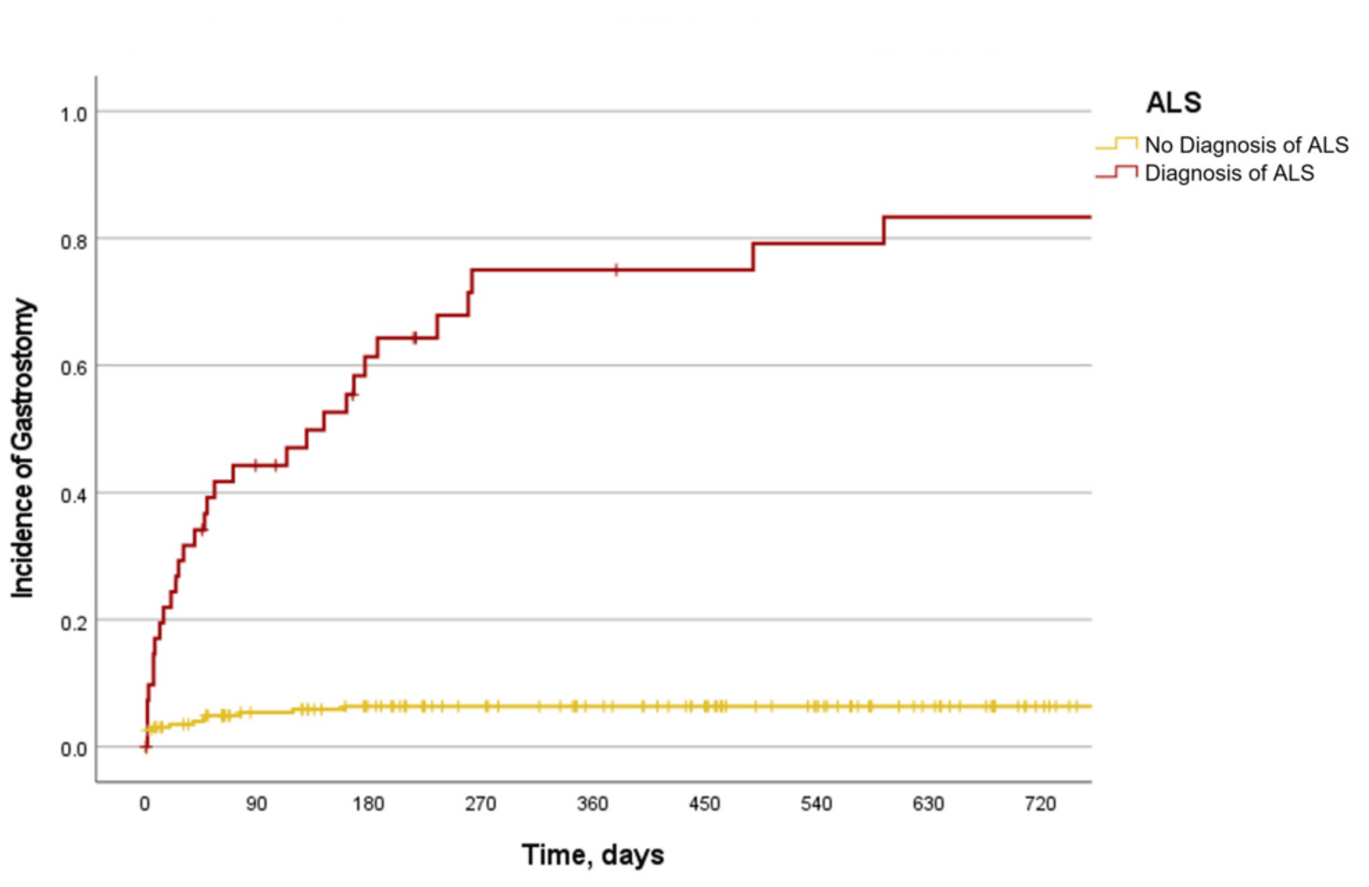
Incidence of gastrostomy placement by diagnosis of ALS The Kaplan-Meier curve of the incidence of gastrostomy placement by diagnosis of ALS. Log-rank P-value: <0.001. ALS: amyotrophic lateral sclerosis

**Figure 5:**
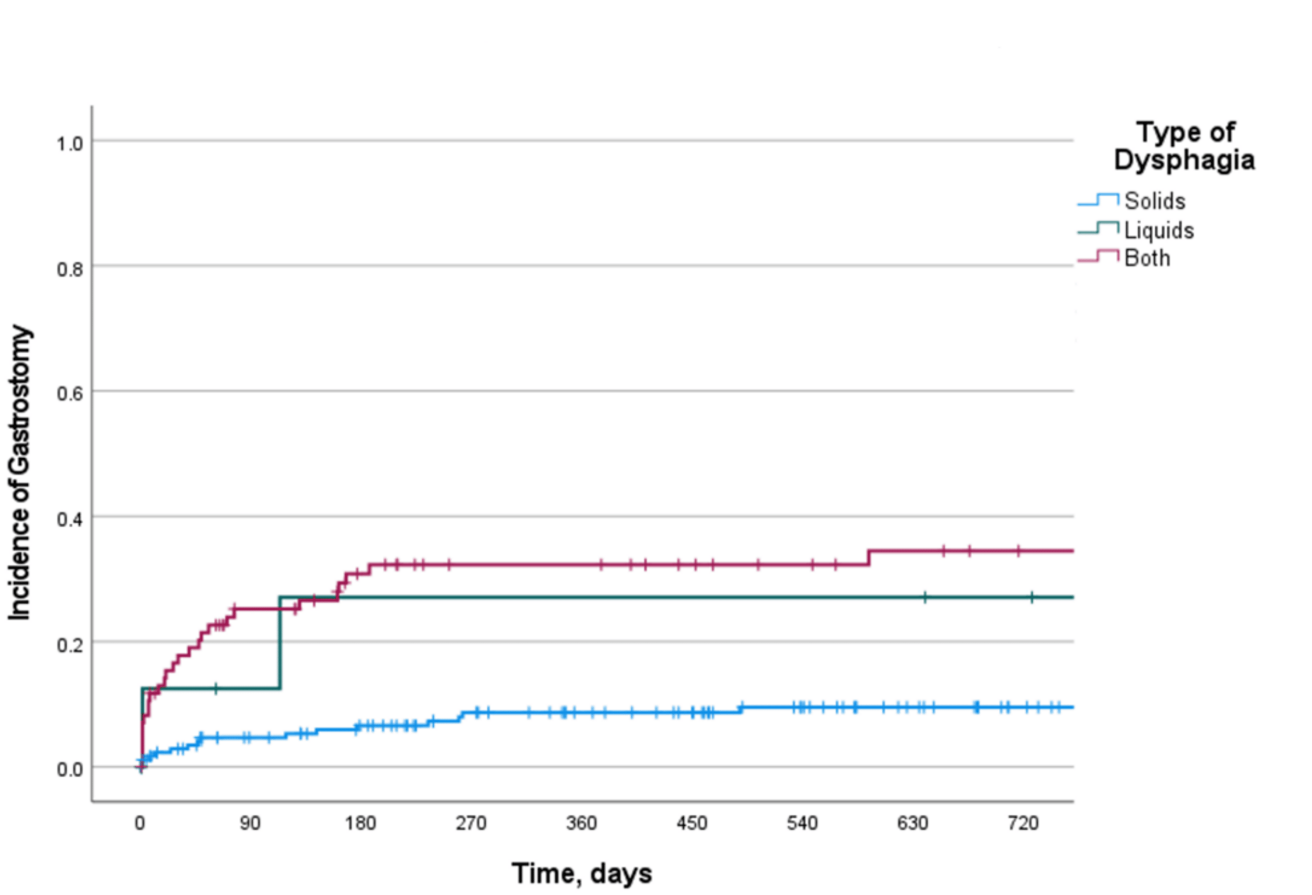
Cumulative incidence of gastrostomy placement by type of dysphagia The Kaplan-Meier curve of the incidence of gastrostomy placement by type of dysphagia. Log-rank P-value: <0.001.

**Figure 6:**
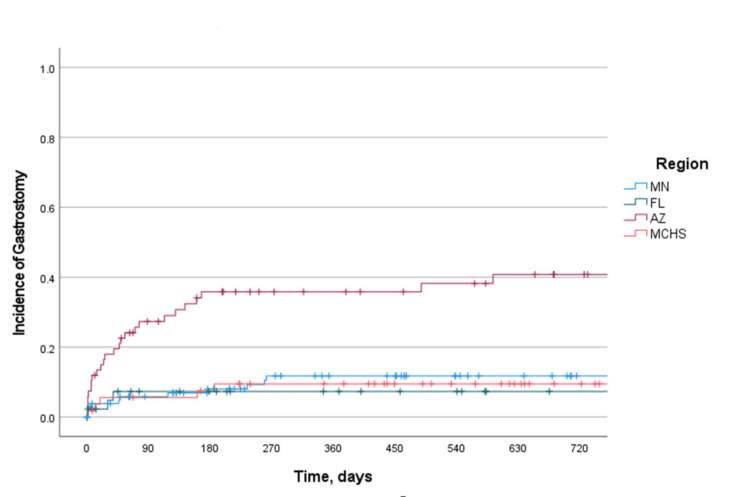
Cumulative incidence of gastrostomy placement by hospital site The Kaplan-Meier curve of the incidence of gastrostomy placement by hospital site. Log-rank P-value: <0.001. MN: Minnesota; FL: Florida; AZ: Arizona; MCHS: Mayo Clinic Health System

In univariable analysis, gastrostomy placement was associated with multiple variables. The strongest associations for gastrostomy placement were BMI, hospital site of evaluation, diagnosis of ALS, diagnosis of MS, CFS score, presence of arm weakness, use of portable ventilator at home, type of dysphagia, and being referred by a neurologist (Table 5 and Figures 4-6).

**Table 5:** Baseline characteristics by gastrostomy placement ^a^Other conditions included lateral sclerosis (n=1), hereditary myopathy (n=8), and critical illness myopathy (n=6). Data reported as No. (%) unless otherwise indicated. ASA: American Society of Anesthesiologists; BMI: body mass index; CCI: Charlson comorbidity index; CFS: clinical frailty scale; EMG: electromyography; ND: neurologic disorder; PFT: pulmonary function test

Demographics	Gastrostomy not performed (n=222)	Gastrostomy performed (n=51)	P-value
Age at evaluation, median (IQR), year	63.4 (55.5-71.7)	67.7 (60.0-73.9)	<0.03
Sex, male	85 (38.3)	31 (60.8)	<0.01
Race, White	204 (91.9)	46 (90.2)	0.78
BMI, median (IQR), kg/m^2^	28.2 (24.1-33.0)	24.7 (21.0-29.2)	<0.01
Site of evaluation			<0.01
Minnesota	94 (42.3)	15 (29.4)	-
Florida	39 (17.6)	4 (7.8)	-
Arizona	41 (18.5)	26 (51.0)	-
Health system	48 (21.6)	6 (11.8)	-
Amyotrophic lateral sclerosis	11 (5.0)	31 (60.8)	<0.01
Myasthenia gravis	58 (26.1)	3 (5.9)	<0.01
Hereditary neuropathy	38 (17.1)	1 (2.0)	<0.01
Inflammatory myositis	21 (9.5)	1 (2.0)	0.09
Inclusion body myositis	15 (6.8)	5 (9.8)	0.55
Multiple sclerosis	71 (32.0)	3 (5.9)	<0.01
Other conditions^a^	8 (3.6)	7 (13.7)	0.01
CFS score, median (IQR)	3 (3-5)	5 (4-7)	<0.01
ASA physical status classification system score, median (IQR)	3 (2-3)	3 (3-4)	<0.01
CCI score, median (IQR)	3 (1-4)	3 (2-4)	0.63
Arm weakness	71 (32.0)	36 (70.6)	<0.01
Leg weakness	75 (33.8)	30 (58.8)	<0.01
EMG performed	87 (39.2)	33 (64.7)	<0.01
Aspiration pneumonia	34 (15.3)	15 (29.4)	<0.03
Speech swallow evaluation	74 (33.3)	25 (49.0)	0.05
Obstructive sleep apnea	87 (39.2)	18 (35.3)	0.64
Portable ventilator	3 (1.4)	12 (23.5)	<0.01
PFTs performed	57 (25.7)	21 (41.2)	0.04
Abnormal PFTs	29 (50.9)	13 (61.9)	0.45
Diabetes	47 (21.2)	4 (7.8)	<0.03
Time with ND, median (IQR), days	1,173 (1-4,790)	169 (−24-799)	<0.01
Type of dysphagia			<0.01
Solids only	160 (72.1)	19 (37.3)	-
Liquids only	6 (2.7)	2 (3.9)	-
Both	56 (25.2)	30 (58.8)	-
Referred by a neurologist	26 (11.7)	17 (33.3)	<0.01

In unadjusted Cox proportional regression analysis, the strongest predictors for gastrostomy placement included diagnosis of ALS, CFS score, ASA classification score, arm weakness, need for portable ventilator at home, and dysphagia to both solids and liquids. After adjusting for age, diagnosis of ALS, CFS score, and dysphagia type, BMI (HR: 0.92 per 1 kg/m^2^; P=0.0092), diagnosis of ALS (HR: 10.39; P<0.0001), CFS score (HR: 1.49 per 1 point; P<0.0001), ASA classification score (HR: 2.23 per 1 point; P=0.0046), and dysphagia to both solids and liquids (HR: 2.57 relative to solids; P=0.0014) were strongly associated with gastrostomy placement (Table 6). Age, male sex, history of aspiration pneumonia, use of portable ventilator, Charlson comorbidity index, and time with ND were not associated with gastrostomy placement.

**Table 6:** Cox proportional hazards regression analysis for gastrostomy placement at follow-up ^a^A total of 51/273 patients had gastrostomy at follow-up. Given the outcome with the lowest event rate (n=51), the maximum number of variables allowed in the multivariable analysis was five. Multivariable Cox proportional hazards regression models were adjusted for the strongest variables associated with gastrostomy at P<0.05 on univariable regression analysis. ^b^The three strongest variables adjusted in the baseline model were amyotrophic lateral sclerosis, CFS score, per 1 point, and dysphagia type. Age at evaluation was added to the baseline four-variable model as a covariable. ASA: American Society of Anesthesiologists; BMI: body mass index; CCI: Charlson comorbidity index; CFS: clinical frailty scale; EMG: electromyography; HR: hazards ratio; NA: not applicable; ND: neurologic disorder; PFT: pulmonary function test

Demographics	Univariable		Multivariable^a^	
	HR (95% CI)	P-value	HR (95% CI)	P-value
Age at evaluation, per 1 year	1.04 (1.01-1.06)	<0.01	1.02 (0.99-1.04)	0.28^b^
Sex, male	2.60 (1.46-4.62)	<0.01	1.30 (0.70-2.42)	0.41
Race, White	0.71 (0.28-1.78)	0.46	1.32 (0.51-3.42)	0.57
BMI, per 1 kg/m^2^	0.92 (0.87-0.96)	<0.01	0.92 (0.87-0.98)	<0.01
Site of evaluation, Arizona	3.62 (2.08-6.29)	<0.01	1.16 (0.60-2.23)	0.66
Amyotrophic lateral sclerosis	17.70 (9.60-32.60)	<0.01	10.39 (5.34-20.19)	<0.01^b^
Myasthenia gravis	0.20 (0.06-0.65)	<0.01	0.63 (0.18-2.16)	0.46
Hereditary neuropathy	0.11 (0.02-0.80)	<0.03	0.27 (0.04-2.01)	0.20
Multiple sclerosis	0.16 (0.05-0.48)	<0.01	0.45 (0.13-1.56)	0.21
CFS score, per 1 point	1.92 (1.61-2.30)	<0.01	1.49 (1.23-1.81)	<0.01^b^
ASA physical status classification system score, per 1 point	3.76 (2.32-6.10)	<0.01	2.23 (1.28-3.89)	<0.01
CCI score, per 1 point	1.03 (0.92-1.17)	0.59	0.99 (0.80-1.22)	0.91
Arm weakness	4.22 (2.31-7.70)	<0.01	0.94 (0.43-2.04)	0.88
Leg weakness	2.62 (1.50-4.57)	<0.01	1.23 (0.67-2.27)	0.51
EMG performed	3.01 (1.68-5.37)	<0.01	2.06 (1.10-3.82)	<0.02
Aspiration pneumonia	2.05 (1.12-3.76)	<0.02	0.95 (0.51-1.78)	0.87
Speech swallow evaluation	2.13 (1.21-3.75)	<0.01	0.91 (0.49-1.68)	0.75
Portable ventilator	12.75 (6.46-25.13)	<0.01	1.66 (0.79-3.47)	0.18
PFTs performed	2.32 (1.32-4.08)	<0.01	0.74 (0.39-1.40)	0.35
Diabetes	0.36 (0.13-1.00)	0.05	0.50 (0.17-1.42)	0.19
Time with ND, per 365 days	0.95 (0.91-0.99)	<0.02	0.99 (0.94-1.04)	0.70
Type of dysphagia				
Solids only	1.00 (reference)	NA	1.00 (reference)	NA
Liquids only	2.82 (0.66-12.15)	0.16	2.73 (0.64-11.63)	0.17
Both	3.97 (2.23-7.07)	<0.01	2.57 (1.44-4.57)	<0.01^b^
Referred by a neurologist	3.19 (1.71-5.97)	<0.01	0.83 (0.40-1.71)	0.62

## Discussion

We found that an ALS diagnosis and referral to a gastroenterologist by a neurologist were negative predictors of EGD recommendation after initial consultation. In contrast, a diagnosis of MG or MS was highly associated with a recommendation for EGD, while there was no significant association with hereditary myopathy, inflammatory myositis, or inclusion body myositis. The differences in recommendation for or against EGD depending on the underlying disease process are likely related to its typical trend of progressive severity and known level of association with dysphagia. For example, conditions like ALS are rapidly progressive and associated with severe neurologic deficits that do not improve, whereas symptoms of MG and MS may wax and wane with comparatively less severe symptoms [20-22]. This is supported by our finding that patients with symptoms associated with greater severity, such as the presence of leg or arm weakness, higher CFS scores, use of a portable ventilator, previous order for pulmonary function tests, and previous speech swallow evaluation, received a recommendation for EGD by the consulting gastroenterologist.

Further, referral to a gastroenterologist by a neurologist was significantly associated with a recommendation against EGD for the evaluation of dysphagia. This is most likely due to the previous diagnosis of the ND responsible for the development of dysphagia. Of all the patients who received an EGD, 50.7% had an abnormality (including esophageal candidiasis, reflux esophagitis, eosinophilic esophagitis, or stricture), 26.9% required an intervention, and 46.3% had a biopsy taken. However, it should be noted that 16.7% of the interventions performed with EGD were urgent or emergent food bolus removals, which is more likely to represent a clinical sequela of advanced neurologic dysphagia (Table 2). Therefore, it follows that those with less severe neurologic symptoms should be evaluated with EGD because a mechanical etiology of dysphagia may be superimposed on a patient who also has an ND. In contrast, for those with more advanced clinical signs of neurologic disease, an EGD is less likely to provide additional benefit aside from intervening on food bolus impaction.

To our knowledge, our study is the first to generate a predictive model for the recommendation of EGD in patients with NDs using the associations we identified (Figure 2, Figure 3). With the exception of sex, the six predictor variables corresponded to either increasing disease severity or increased disease duration. It is our hope that our predictive model will enhance decision-making on dysphagia workup in future patients with NDs.

As reflected in our model, those diagnosed with ALS who had been referred by a neurologist may have had more clinical certainty of the diagnosis and therefore a greater need for gastrostomy placement rather than EGD. This is supported by our finding that ALS was the greatest risk factor for gastrostomy tube placement. In ALS, we found that most gastrostomy placements occurred within one year of evaluation by a gastroenterologist (Figure 4). Other than this diagnosis, the presence of solid and liquid dysphagia was the main clinical finding that predicted gastrostomy tube placement (Figure 5). Notably, this same variable was predictive of a recommendation against EGD. This ultimately suggests a bimodal approach in which patients with less severe symptoms and a nonterminal ND diagnosis may receive EGD, while those with severe symptoms or a waxing and waning ND may benefit from gastrostomy tube placement. This is supported by our finding that higher scores on both the ASA classification and CFS were highly predictive of the need for gastrostomy tube placement. At the initial gastroenterology consultation, 13.9% of patients with NDs were given a recommendation for gastrostomy. However, over the 10-year retrospective window, a total of 18.7% of patients with NDs would ultimately receive a gastrostomy (Table 3). This finding identifies a proportion of patients who will eventually develop a need for gastrostomy, which will require a high level of clinical suspicion on behalf of the consulting gastroenterologist over time. Anticipation of the need for gastrostomy is underrepresented in the medical literature but remains a core component of nutritional support for patients with NDs [23].

We suggest that when considered in conjunction with the predictive clinical feature of dysphagia to both solids and liquids, these scores may help clinicians anticipate gastrostomy tube placement when applied to NDs. Ultimately, the decision to pursue gastrostomy placement, whether endoscopically or radiologically, is accompanied by an inherent risk of increased morbidity and mortality that can vary from 2.4% to 23.5% [24].

Our study had several strengths, including the geographically diverse and multisite nature of our patient cohort. Patients included in the study were seen at large tertiary care centers located in the Southwest, Midwest, and Southeast regions of the United States. Interestingly, we found that slightly more gastrostomies were placed in the Southwest region, which may reflect a pattern of practice and preferential referral of patients with more advanced levels of clinical severity to a specific clinical site as is commonplace in our institution (Figure 6). We evaluated multiple markers of ND and dysphagia severity that encompassed subjective symptom scores, imaging, neurologic testing, swallow studies, manometry, and physical examination findings.

Our study was limited by its 10-year duration and retrospective nature. Although 10 years is adequate for studying dysphagia in more rapidly progressive neurologic conditions, slower-moving conditions may not be adequately assessed. This is reflected in our finding that most gastrostomy placements in patients with NDs occur within the first year after evaluation by a gastroenterologist for dysphagia (Figure 1). NDs with slower progression may benefit from further prospective evaluation including further application of our proposed predictive model.

## Conclusions

When considering gastrostomy placement, ALS was the greatest risk factor, with most patients receiving gastrostomy within one year of gastroenterology consultation. Of all patients with NDs seen at the initial gastroenterology consultation, 13.9% were recommended gastrostomy, and an additional 18.7% would receive a gastrostomy over the next 10 years. Identification of predictors of gastrostomy need for this large proportion of patients with NDs provides a critical window for the optimization of ND management before the need for gastrostomy develops. The diagnosis of ALS, the presence of solid and liquid dysphagia, and high CFS and ASA classification scores are independent predictors of the need for gastrostomy placement. When considering EGD in patients with NDs, patients referred to gastrointestinal (GI) by a neurologist and patients with rapidly progressive NDs, like ALS, displayed more severe symptoms and were more likely to receive a recommendation for EGD by the consulting gastroenterologist. Given the complex interplay of risk factors, we propose a predictive model for EGD recommendation in the evaluation of dysphagia secondary to ND. We hope that this model can assist clinicians in their medical decision-making process, particularly in making diagnostic decisions for this complex patient population.

Both the prediction of EGD and gastrostomy recommendation require a high degree of clinical suspicion and recognition of a complex interplay of independent risk factors. Now, with artificial intelligence (AI) rapidly being incorporated into medical education, the electronic health record, and clinical decision-making, there is an opportunity to apply the predictive findings of this study to machine learning tools. In the future, our proposed model and findings would be best deployed as an AI clinical decision-making tool that will augment the clinical suspicion and awareness of a need for EGD or gastrostomy in patients with NDs.
